# Transcranial Alternating Current Stimulation (tACS) Does Not Affect Sports People’s Explosive Power: A Pilot Study

**DOI:** 10.3389/fnhum.2021.640609

**Published:** 2021-04-29

**Authors:** Andreina Giustiniani, Giuseppe Battaglia, Giuseppe Messina, Hely Morello, Salvatore Guastella, Angelo Iovane, Massimiliano Oliveri, Antonio Palma, Patrizia Proia

**Affiliations:** ^1^IRCCS San Camillo Hospital, Venice, Italy; ^2^NEUROFARBA Department, University of Florence, Florence, Italy; ^3^Sport and Exercise Sciences Research Unit, Department of Psychological, Pedagogical and Educational Sciences, University of Palermo, Palermo, Italy; ^4^Department of Statistics, SEAS, University of Palermo, Palermo, Italy

**Keywords:** brain stimulation, motor cortex, sport performance, polymorphism, BDNF, ACE, tACS

## Abstract

**Purpose**: This study is aimed to preliminary investigate whether transcranial alternating current stimulation (tACS) could affect explosive power considering genetic background in sport subjects.

**Methods**: Seventeen healthy sports volunteers with at least 3 years of sports activities participated in the experiment. After 2 weeks of familiarization performed without any stimulation, each participant received either 50 Hz-tACS or sham-tACS. Before and after stimulation, subjects performed the following tests: (1) the squat jump with the hands on the hips (SJ); (2) countermovement jump with the hands on the hips (CMJ); (3) countermovement jump with arm swing (CMJ-AS); (4) 15-s Bosco’s test; (5) seated backward overhead medicine ball throw (SBOMBT); (6) seated chest pass throw (SCPT) with a 3-kg rubber medicine ball; and (7) hand-grip test. Additionally, saliva samples were collected from each participant. Genotyping analysis was carried out by polymerase chain reaction (PCR).

**Results**: No significant differences were found in sport performance of subjects after 50 Hz-tACS. Additionally, we did not find any influence of genetic background on tACS-related effect on physical performance. These results suggest that tACS at gamma frequency is not able to induce an after-effect modulating sport performance. Further investigations with larger sample size are needed in order to understand the potential role of non-invasive brain stimulation techniques (NIBS) in motor performances.

**Conclusions**: Gamma-tACS applied before the physical performance fails to improve explosive power in sport subjects.

## Introduction

Electroencephalographic recordings have shown a functional role of brain oscillations in motor execution of the body’s movements. In particular, in the motor cortex (M1), beta oscillations (13–30 Hz) seem to be prevalent during rest, while gamma rhythm (>30 Hz) synchronizes when subjects focus attention (Jensen et al., 2007) and during voluntary movement preparation (Murthy and Fetz, 2006; Sanes and Donoghue, 2006) and execution (Pfurtscheller et al., 2003). Studies using invasive recordings have provided the general idea that in the central brain regions, gamma band responses are related to externally paced (Pfurtscheller and Lopes da Silva, 1999; Ohara et al., 2000) and self-paced hand and arm movements (Pfurtscheller et al., 2003; Szurhaj et al., 2005; Miller et al., 2007; Ball et al., 2008). The increase in gamma synchronization reflects enhanced cortical excitability (Schroeder and Lakatos, 2009) as well as neural computation of movements’ details (Rickert, 2005). The growing number of studies reporting gamma synchronization during movement execution (Pfurtscheller et al., 2003; Brovelli et al., 2005) suggested that it might be the elective rhythm involved in voluntary movement and that its synchronization may facilitate motor processing.

In the past decades, the functional role of brain oscillations in motor performance has been further investigated using non-invasive brain stimulation (NIBS) techniques. Transcranial magnetic stimulation (TMS) set at 50 Hz has been used to induce changes in cortical excitability, as measured with motor-evoked potentials (MEP) as well as in motor performance (Chen et al., 1997; Oliveri et al., 2005). Among electric stimulation techniques (tES), transcranial direct current stimulation (tDCS) (Paulus, 2011) has been employed to improve performance in several athletes such as cyclists (Okano et al., 2015) and skiers (Reardon, 2016). In particular, after 20 min of anodic tDCS over the left temporal cortex, a significant enhancement of peak power and a reduced effort perception has been found in cyclists during an incremental cycling test (Okano et al., 2015). Such changes have been investigated also in genetic studies (Brunoni et al., 2013; Puri et al., 2015) that have shown an effect of the polymorphism Val66Met in the brain-derived neurotrophic factor (BDNF) gene in the tDCS-induced plasticity in older adults (Puri et al., 2015).

A new electrical stimulation technique, known as transcranial alternating current stimulation (tACS), has been introduced as a promising tool, able to enhance brain oscillations and modulate cognitive (Feurra et al., 2012), perceptual (Kanai et al., 2008, 2010), and motor performances (Pogosyan et al., 2009; Feurra et al., 2011; Giustiniani et al., 2021). The tACS protocols include the application of electrical alternating currents to the scalp through electrodes placed over a target brain region. Such sinusoidal stimulation has been shown to modulate neuronal membrane potential (Ali et al., 2013). tACS is usually delivered at weak intensities (typically between 1 and 2 mA), and depending on the stimulation setting, tACS effects have been shown to outlast the end of the stimulation (i.e., after-effect; Kasten et al., 2016). Previous studies have shown that when applied over M1 at 50 Hz, tACS improves velocity and acceleration of visually triggered movements compared with beta or sham-tACS (Moisa et al., 2016). Interestingly, driving gamma oscillation at 50 Hz in the motor cortex leads to a significant duration-dependent modulation of local GABA_A_ inhibition. Such changes in GABAA during tACS correlate with motor behavior (Nowak et al., 2017) and might be a link between tACS effects and sport performances (Schutter and Hortensius, 2011; Sakashita et al., 2019). Accordingly, in a recent study, we found that tACS at 50 Hz applied over M1 affects motor performance and induce a long-lasting modulation of M1 excitability levels (Giustiniani et al., 2021), similar results are obtained for motor performance when tACS electrodes are placed over the cerebellum (Giustiniani et al., 2021).

As suggested by previous studies, tACS effects may outlast the end of the stimulation (i.e., after-effect; Zaehle et al., 2010; Helfrich et al., 2014). While tACS online effects mostly have been described in terms of entrainment (Helfrich et al., 2014), tACS after-effects, lasting up to 70 min after stimulation’s end (Kasten et al., 2016), have been shown to rely on other mechanisms not strictly related to the entrainment (Moliadze et al., 2010; Vossen et al., 2015; Antal and Herrmann, 2016; Brighina et al., 2019; Giustiniani et al., 2019). In particular, the offline effect of tACS might be dependent on synaptic plasticity. Namely, the BDNF gene mediates the specific effect of tACS in neuronal oscillations (Riddle et al., 2020). Of note, the presence of the Val66Met, plays a role also in mediating the efficacy of NIBS. Indeed, the presence of the Val66Met polymorphism seems to predict reduced efficacy for neuromodulation. When the Val66Met polymorphism is present, a reduced neural response to brain stimulation would be caused by a decreased NMDA-dependent long-term potentiation of synaptic transmission (Antal et al., 2010; Fritsch et al., 2010; Podda et al., 2016). Interestingly, the presence of this polymorphism has been shown to reduce also the beneficial effect of physical activity (Erickson et al., 2013). Overall, this evidence suggests that entrainment and long-lasting tACS-related effects can improve motor skills, such as motor learning and muscular strength inducing optimal performance during sports through the enhancement of synaptic plasticity (Fritsch et al., 2010). Therefore, the genetic background should be taken into account to estimate tACS-induced effect, especially in the context of sports. Unfortunately, to date, no studies have investigated whether tACS effects might be transferred to the real sport performance and whether the genetic background might influence the response to tACS in athletes. Only recently, some authors have coined the term “neurodoping” to indicate the use of these emerging techniques to enhance physical and mental performance in sport (Jenkinson and Brown, 2011; Reardon, 2016; Colzato et al., 2017). While, on one hand, tDCS effects have been investigated during sports performances (Okano et al., 2015; Reardon, 2016), so far, no studies have been conducted using tACS. In the sports domain, to transfer the stimulation effects from a single movement or motor behavior to a motor performance poses an important and ambitious challenge to researchers, and it constitutes a critical aspect in athletes’ technical–tactical acts (Podda et al., 2016).

Therefore, the aim of the present pilot study was to investigate whether modulating gamma rhythm over M1 could improve physical performance in sports subjects when applied prior to sports performance. To this end, we applied 50-Hz tACS or sham-tACS in sports subjects immediately before the execution of a set of physical tests. As gamma oscillations have been found to be involved in force production (Muthukumaraswamy, 2010; Naro et al., 2017), we applied tACS before a set of physical tasks traditionally employed to measure force levels (Glatthorn et al., 2011; Proia et al., 2019; Bonaventura et al., 2020). Additionally, we investigated whether the presence of the angiotensin-converting enzyme (ACE) polymorphism, traditionally related to the force level, mediated the effect of tACS in tasks requiring grip execution (Costa et al., 2009).

Previous studies have shown that female and male athletes showed significant differences in sports performances and physical tests, attributable to sex-specific differences in neuromuscular control and in body mass (Battaglia et al., 2014; McFarland et al., 2016; Holden et al., 2019). Therefore, it might have important implications to explore separately tACS-related sex-specific responses in sports people. Additionally, as it has been shown that genetic background affects individual response to tES (Brunoni et al., 2013; Puri et al., 2015), we evaluated whether the brain-derived neurothrophic factor (BDNF) Val66Met polymorphisms was associated with tACS physical response.

## Materials and Methods

### Participants

Seventeen healthy sports volunteers (7 males and 10 females), aged between 18 and 49 years (27.29 ± 10.65) with at least 3 years of sports activities, were randomly assigned to the real-tACS or sham-tACS group, respectively (see Table 1).

All participants were in good health and provided written informed consent. All participants were naïve to the experimental hypotheses and were right-handed according to the Edinburgh handedness inventory. After having been enrolled in the study, participants were randomly assigned to the real-tACS (four males and seven females) or the sham-tACS group (three males and three females), respectively. Inclusion criteria were age between 18 and 50 years old and training three times per week. Exclusion criteria included brain injury, neurological or psychiatric illness, presence of intracranial metallic plates, cardiac pacemakers, pregnancy, family or personal history of epilepsy, and chronic pain.

**Table 1:** The descriptive information about the participants.

ID	BDNF	ACE	AGE	Group	Height	Weight
1	AG	DD	18	Real tACS	1.67	68
2	GG	DD	38	Real tACS	1.65	71
3	GG	ID	19	Real tACS	1.63	48
4	AG	ID	28	Real tACS	1.78	83
5	GG	DD	47	Real tACS	1.65	70
6	AG	DD	46	Real tACS	1.7	69
7	AG	ID	23	Real tACS	1.63	50
8	AG	DD	23	Real tACS	1.84	90
9	AG	DD	22	Sham	1.68	68
10	AG	DD	23	Sham	1.65	63
11	GG	DD	20	Sham	1.80	69
12	GG	DD	21	Real tACS	1.65	62
13	AG	DD	21	Sham	1.8	120
14	AG	DD	19	Real tACS	1.70	83
15	AG	DD	20	Real tACS	1.60	48
16	AA	DD	49	Sham	1.57	55.6
17	AG	DD	27	Sham	1.75	69

*Note. During the experimental session, an informal interview was conducted and descriptive information as well as information about the height and the weight were collected*.

Participants were instructed to refrain from consuming caffeine or analgesic medications or engaging in vigorous physical activity for at least 24 h prior to the experimental sessions. Adherence to these instructions was confirmed on arrival. The study was in compliance with the Helsinki declaration and approved by the Ethical Board of the University of Palermo.

### Experimental Design

**Figure 1:**
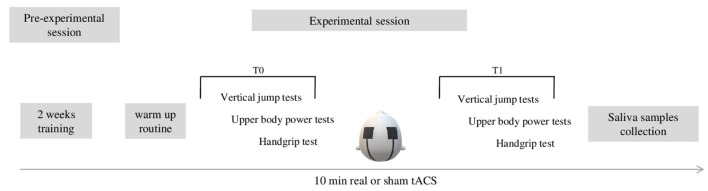
The figure shows the experimental procedure. Before the experimental session, subjects performed 2 weeks (two sessions/week) of training. The experimental session lasted approximately 1 h and included 2–3 min of warm up routine consisting of light jogging, 20 min of execution of the experimental physical tests (t0), 10 min of real or sham-tACS, and a new assessment of performance of physical tests (20 min). Afterward, saliva samples were collected from each participant.

In the present double blind, sham-controlled pilot study, we investigated the effects of tACS on upper and lower limbs explosive strength. To explore tACS-related effects, a set of physical tests was administered before and after either real or sham stimulation, in a between-subjects design (Figure 1). Participants were enrolled in the study 2 weeks before the experimental session. In a preliminary session, subjects underwent an interview in order to collect personal data such as age, competition level, and years of sports practice. In this preliminary session, they were instructed to not perform any training for at least 48 h before the experimental performance. During the 2 weeks preceding the experimental session, participants performed a familiarization (two sessions/week) with the standard procedure of the physical tests in agreement with Duncan and Moir (Moir et al., 2004; Duncan et al., 2005). During the experimental session, participants performed: (1) anthropometric measurements; (2) a warm-up routine with 2–3 min of light jogging (Nowak et al., 2017); (3) baseline measurement at physical tests (T0); (4) real or sham tACS; and (5) post-stimulation measurement at physical tests (T1). A saliva sample was collected from each participant in order to perform genetic analysis. In particular, polymorphisms in ACE and BDNF genes were analyzed. Each session lasted for approximately 1 h and included a brief informal interview, a warm up routine, 20 min of physical tests followed by 10 min of tACS. After the stimulation’s end, subjects were asked to perform again the same physical tests as before the stimulation.

### tACS

**Figure 2:**
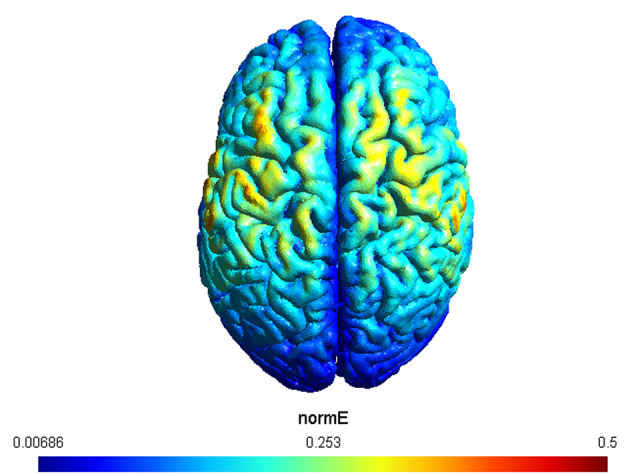
The figure depicts the simulation of the applied electric fields. Electrodes were placed over C3 and C4, respectively. The maximum strength of the normalized electric field is color coded from 0.006–0.5 mV/mm.

A double-blind procedure was used so that, before the experimental session, a second experimenter set the mode (e.g., real or sham) on the stimulator, and did not interact with the subject or experimenter who performed data collection. tACS was delivered using a DC stimulator (Brainstim, EMS) through a pair of saline-soaked sponge electrodes (5 × 5 cm^2^). Electrodes were placed over the left and right motor cortices (C3 and C4 according to the international 10–20 EEG international placement system). This electrode montage was chosen since it has been shown to modulate excitability levels of both left and right motor cortices (Heise et al., 2019), as required by the experimental physical tests. In addition, we simulated the expected current distribution over the brain tissue underlying the electrodes by using the SIMNIBS 3 software (Saturnino et al., 2018). The result of the simulation, visualized using GMSH, confirmed the left and right motor cortices as the target regions (Figure 2).

tACS was applied at 50 Hz for 10 min, before the motor trials, with a current intensity of 1.5 mA peak-to-baseline (corresponding to 0.06 mA/cm^2^ current density under each electrode). Stimulation waveform was sinusoidal without DC offset. The current was ramped up over the first 30 s of stimulation. The control group underwent sham tACS, where tACS was pre-programmed to interrupt after 15 s in order to elicit the typical tingling sensation under the electrodes at the beginning of the stimulation, without affecting underlying brain rhythms. Stimulation was in compliance with established safety protocols regarding DC and AC stimulation (Matsumoto and Ugawa, 2017). Subjects were blind to experimental hypothesis and stimulation type.

### Anthropometry

Height was measured using a standard stadiometer (maximum height recordable at 220 cm; resolution at 1 mm) with the subjects barefoot and standing upright. Body weight was measured using a Seca electronic scale (maximum weight recordable at 300 kg; resolution at 100 g; Seca, Hamburg, Germany) with the subjects wearing only underwear.

### Warm-Up Routine

Before the experimental performance, in agreement with Duncan et al. (2005), all participants followed a warm-up routine consisting of 2–3 min of light jogging. In order to avoid possible negative effects on the vertical jump, no stretching exercises were carried out (Sakashita et al., 2019).

Fifteen minutes after the warm-up period, subjects were asked to start the physical test execution. A 2 min passive recovery occurred between the end of the warm-up and the beginning of the tests for restoration of ATP-PC stores (Duncan et al., 2005).

### Physical Tests

The following tests were performed: the squat jump with the hands on the hips (SJ), countermovement jump with the hands on the hips (CMJ), countermovement jump with arm swing (CMJ-AS), 15-s Bosco’s test, seated backward overhead medicine ball throw (SBOMBT), and seated chest pass throw (SCPT) with a 3-kg rubber medicine ball (Battaglia et al., 2014). All tests were performed three times, but only the best performance was taken into account for the subsequent analysis. The exercises’ sequence was kept constant throughout all the repetitions within and between sessions.

#### Vertical Jump Tests

Participants were instructed to perform each test with maximal effort. They performed four jump tests. All jumps were executed on an optoelectronic platform (Optojump, Microgate S.R.L., Italy) transmitting an infrared light 1–2 mm above the floor so that, when the light was crossed by the feet, the units triggered a timer with a precision of 1 ms, which allowed the measurement of flight time and contact time, respectively (Glatthorn et al., 2011).

In the SJ, subjects started in a half squat position with their hands on their hips. For the CMJ, subjects performed a single jump using a countermovement from standing upright with their hands on their hips throughout the entire jumps. During the CMJ-AS, participants performed a single vertical jump using a countermovement from the standing upright with the arms down the sides (Duncan et al., 2005). Finally. in the 15-s Bosco’s test, participants were instructed to jump as high as possible, while trying to retain short ground contact times and keeping the hands on the waist (Carlock et al., 2004). The mean power during the 15-s tests was recorded in W·kg^−1^.

#### Upper Body Power Tests

To evaluate the upper body power, subjects were asked to sit on a chair (35 cm length, 35 cm breadth, 42 cm height). A tape for measure was located on the floor and stretched out to the distance of 10 m. Subjects were invited to sit with their back against the chair and their feet flat on the ground. They were then asked to hold the ball using both the hands and to extend the arm away from the chest in order to consider their different arm lengths. Finally, subjects were instructed to drop the ball straight down on to the tape measure.

During SCPT, subjects were instructed to powerfully push the ball away from the center of their chest as far as possible. During SBOMBT, subjects, with shoulders flexed to 90° with totally extended elbows and ball in their hands, threw the ball vigorously backward over their heads. The measurement was considered valid and recorded only when the front of the ball hit the measuring tape (Glatthorn et al., 2011; Harris et al., 2011). The exercise was repeated three times interleaved by 2 min of rest.

#### Handgrip Test

The standardized procedure for maximal hand grip strength task is the following: while standing barefoot, feet at shoulders’ width apart and head in neutral position gazing forward, arms extended laterally alongside the trunk, participants were instructed to exert maximum force on the handgrip dynamometer (Kern Map model 80K1-Kern^®^, Kern & Sohn GmbH, Balingen, Germany). Participants were not allowed to make any other ancillary bodily movements during the handgrip task. Each participant exerted 3 s of maximal isometric force with each hand, alternating the right and the left hand, for a total of three trials with a 3 min rest between trials. Trials were scored as the maximal isometric strength expressed in kgf units, using the best performance out of the three for data analysis.

### Sample Collection and Genotyping

As described in Puri et al. (2015), saliva samples were collected in 15-ml sterile tubes, and the subjects were instructed to fast in the 3 h prior to the experiment. Each sample was appropriately stored at −20°C until use for analyses. Genomic DNA was isolated from 1 ml of whole saliva using a PureLink blood kit (PureLink Genomic DNA Thermo Fisher Scientific). The genotyping was carried out by polymerase chain reaction (PCR) in a total reaction volume of 50 μl containing 50 ng of template, 1 μl of 10 mM deoxynucleoside triphosphate (dNTPs), 1 μl of 30 pmol each primer, and 5 μl of 10× reaction buffer with MgCl_2_. The target sequence was amplified using a 5 U/μl Dream Taq (Thermo Fisher Scientific) and the primers were P1 (forward) 5′-CCTACAGTTCCACCAGGTGAGAAGAGTG-3′; P2 (reverse) 5′-TCATGGACATGTTTGCAGCATCTAGGTA-3′; P3 (G allele-specific reverse) 5′-CTGGTCCTCATCCAACAGCTCTTCTATaAC-3′; P4 (A allele-specific forward) 5′-ATCATTGGCTGACACTTTCGAACcCA-3′ used to determine the BDNF genotype and 5′-CTG GAG ACC ACT CCC ATCCTT TCT-3′ (forward) and 5′-GAT GTG GCC ATC ACA TTCGTC AGA-3′ (reverse) used to determine the ACE genotype. PCR amplification was performed with the following protocol: denaturation at 94°C for 5 min, followed by 35 cycles of denaturation at 94°C for 30 s, annealing at 62.5°C for 60 s for BDNF, and 66°C for 30 s for ACE, extension at 72°C for 30 s, and final extension at 72°C for 5 min. The fragments were separated on 8% vertical polyacrylamide gel at 100 V for 1 h and visualized with ethidium bromide.

### Statistical Analysis

Data were statistically analyzed using R (Team, R., 2015). Gaussian distribution was tested using the Shapiro–Wilk test. Bartlett’s test was used to test homoscedasticity. Statistical significance of the main effect was evaluated through F-test. To evaluate differences in physical performance, adjusting for gender and genetic variables, repeated measure of ANOVA was performed for each exercise with time (T0 vs. T1), stimulation (sham-tACS vs. real-tACS), gender (male vs. female), ACE polymorphism [ID vs. DD (none of the participants had II genotype)], and BDNF polymorphism (AA vs. AG vs. GG) as between-subject factors. The interaction time × stimulation was also explored. Tukey’s honest significant difference (HSD) *post hoc* test was conducted to test the main effects and interactions when appropriate.

## Results

### Physical Tests

Table 2 shows the mean and standard deviation obtained in each test, for each group before and after tACS, respectively. ANOVA revealed no significant differences between the two groups before and after the real and sham-tACS.

**Table 2:** The mean values and standard deviation at times 0 and 1 for real and sham-tACS group, for each physical test.

Physical tests	Sham-tACS		Real-tACS	
	Time 0	Time 1	Time 0	Time 1
SCPT (m)	3.59,0.98	3.56,0.81	3.68,0.99	3.65,0.90
SBOMBT (m)	4.57,0.72	4.44,1.07	4.60,1.11	4.70,1.10
Squat jump (cm)	25.90,4.12	25.33,3.10	24.98,8.28	24.20,8.32
Counter movement jump (cm)	26.58,3.70	26.07,3.20	26.68,9.68	25.40,8.89
Counter movement jump arm-swing (cm)	30.80,3.51	29.47,4.36	30.71,9.22	29.05,9.51
Bosco 15 s—ground contact time (s)	0.22,0.04	0.21,0.03	0.22,0.03	0.22,0.03
Bosco 15 s—mean height (cm)	14.88,3.05	13.70,3.44	9.40,3.12	9.80,2.53
Handgrip right hand (kg)	35.52,11.87	35.17,13.09	40.25,12.38	40.91,12.34
Handgrip left hand (kg)	36.55,12.31	35.42,14.02	40.85,11.13	40.15,10.44

As expected, we found a significant difference between men and women in all physical tests, except for the Bosco 15-s ground contact time. In particular, a significant difference emerged in: (1) SBOMBT (diff = 1.494, *p* < 0.001); (2) SCPT (diff = 1.345, *p* < 0.001); (3) handgrip left hand (diff = 17.008, *p* < 0.001) and right hand (diff = 17.026, *p* < 0.001); (4) SJ (diff = 6.486, *p* = 0.002); (5) CMJ (diff = 6.082, *p* = 0.010) and (6) CMJ-AS (diff = 6.240, *p* = 0.006). Tukey HSD confirmed that, generally, males performed better than females (all *p*s < 0.01).

### Genotyping

Regarding BDNF genotyping, the AA genotype was found in one subject that was in the sham group, while GG genotype was present in four subjects of the real-tACS group and in one subject of the sham group. The AG genotype was present in seven subjects of the real-tACS group and in four subjects that underwent the sham tACS. This unbalanced distribution of the genes in the sample suggests to carefully consider the possible generalization of the results. A significant effect of ACE was found for SJ (*p* = 0.004), CMJ (*p* = 0.002), and CMJ-AS (*p* = 0.001), while BDNF had no significant effect. Subjects with ID genotype in ACE gene had, on average, lower performance as shown by the Tukey HSD (*p* = 0.007, *p* = 0.004, *p* = 0.003, respectively). No significant interaction time × stimulation was observed. Table 3 reports all the *p*-values.

**Table 3:** The effect (*p*-value) of gender, age, angiotensin-converting enzyme (ACE), and brain-derived neurotrophic factor (BNDF) in each physical test.

Physical tests	Gender	Age	ACE	BDNF
SBOMBT	<0.001	0.19	0.398	0.198
SCPT	<0.001	0.48	0.457	0.288
SJ	<0.002	<0.001	0.004	0.952
CMJ	<0.010	<0.001	0.002	0.893
CMJAS	<0.006	<0.001	0.001	0.178
BOSCO GC	<0.067	<0.001	0.206	0.528
BOSCO MH	<0.090	0.68	0.697	0.110
HG R	<0.001	0.13	0.217	0.420
HG L	<0.001	0.06	0.483	0.340

*Note. Significance level was set at *p* < 0.05*.

## Discussion

The aim of this pilot study was to investigate the effect of tACS on physical performance in a group of sport subjects. The preliminary results showed that tACS does not modulate sport performance when applied immediately before it. Although they should be interpreted cautiously due to the small sample size, our results suggest that the lack of tACS effects does not depend on the genetic background of the subjects. To the best of our knowledge, this is the first study investigating the role of tACS in athlete subjects taking into account how genetic background may affect individual response to stimulation. In particular, the novelties of this pilot study were to investigate whether genetic background may influence response to tACS in physical exercise and to apply tACS on motor performance in real-life context.

To date, there has been a small amount of research looking at the motor effects of 50-Hz tACS. All of these studies have been conducted in a laboratory context. The lack of evidence reporting the real-life effect of tACS allows only a partial comparison of our results with previous studies. Despite some studies having reported no changes in motor performance during and after gamma-tACS (Miyaguchi et al., 2018), other studies have found an after-effect in motor behavior after gamma tACS (Joundi et al., 2012; Pollok et al., 2015). In particular, Joundi et al. (2012) reported an improvement of grip force during stimulation. Differences in the results might be due to the mismatch in the applied frequencies (70 Hz in the study of Joundi vs. 50 Hz in our study) as well as in the electrode montage (left M1 and the ipsilateral shoulder vs. left and right M1) and electrode size (5 × 7 cm^2^ and 5 × 10 cm^2^ vs. 5 × 5 cm^2^). Divergent results might also be explained by the cortical network state dependence of tACS effects (Kutchko and Fröhlich, 2013; Nowak et al., 2017). However, the main difference between previous findings and our preliminary results lays in the application of tACS immediately before motor performance rather than during it. Since it has been shown that different mechanisms might account for tACS online and offline effects, stimulating before the performance might have led to a lack of effect. Overall, these findings suggest the need for well-controlled tACS protocols, which should be ideally based on accurate electric field predictions (Ali et al., 2013). Indeed, when applying tACS, finding online effects does not strictly imply that these effects will outlast the end of the stimulation. At a first glance, the lack of offline effects suggests that 50-Hz tACS applied with this interhemispheric montage does not induce any long-term effect on motor performance. However, it might well be that tACS has induced after-effects that we were not able to detect with our experimental paradigm (i.e., changes in MEP amplitudes). Indeed, as pilot investigation, we applied tACS for the first time in daily living complex tasks rather than in an experimental task that is able to isolate a single variable of interest.

Additionally, since we applied 50-Hz tACS at rest, it is possible to hypothesize that neural networks did not oscillate at this frequency band during rest but only during movement’s execution. Therefore, another possible explanation is that entrainment of brain oscillations did not occur, simply because groups of neurons oscillated at higher or lower frequency bands (Helfrich et al., 2014). In other words, applying gamma tACS while subjects were resting might have caused a failure of entrainment. In this line of reasoning, it is conceivable to hypothesize that applying tACS during the task might have a different effect on sports performance. Future studies are needed to understand the effect of tACS applied during real-life physical performance.

However, we should acknowledge that some methodological points might account for the null results. Indeed, to modulate gamma activity over the left and the right motor cortex, we used an inter-hemispheric montage that is not the most commonly used montage to affect motor cortex. The choice of this montage was made in order to affect both C3 and C4 because the performed tasks, executed using both hands, required a bilateral activation. Although a previous study reported an effect in terms of changes in excitability levels after the application of tACS with this montage (Schutter and Hortensius, 2011), we cannot exclude that applying the reference electrode over an extracephalic region or over the contralateral frontopolar cortex (above the eyebrow) might have allowed a better targeting of M1. However, the simulation of the electric field suggested a reliable modulation induced over the left and right M1. On the other hand, we found a significant effect of age in the performance at some physical tests. This result is in line with previous studies showing that age is associated with slower performance and altered recruitment of specific brain regions, such as the prefrontal cortices during visuo-motor tasks (Berchicci et al., 2014; Battaglia et al., 2020; Giustino et al., 2020). As easily conceivable, age might affect the physical performance so that the older the subjects, the worst the performance. However, further studies would be needed targeting the effect of age in physical tasks.

Regarding the genetic analyses, our preliminary results did not detect any relationship between the BDNF Val66Met polymorphism and the response to tACS. This result is apparently in contrast with a previous study reporting a greater increase in the power of alpha oscillations after alpha tACS in subjects with the Val66Val rather than in the Val66Met carriers (Riddle et al., 2020). This difference in the results might be due to the different stimulation frequency; indeed, given its role in shaping state-dependent neural excitability, the modulation of alpha oscillations might be more dependent on synaptic plasticity and consequently on BDNF polymorphism (Riddle et al., 2020). Therefore, we cannot exclude that that gamma tACS after-effect might be less dependent on the BDNF polymorphism than the effect of alpha tACS. However, this interpretation should be taken cautiously due to the small sample size of our study; indeed, analysis of genetic polymorphisms in such a small sample may not be representative of the general population (Neuling et al., 2013). On the other hand, studies using tDCS have reported no impact of BDNF polymorphism on response to tDCS (Brunoni et al., 2013). Our results are only partially comparable with the previously reported evidence. Indeed, different forms of current (i.e., direct vs. alternating) might exert different effects at cellular level and therefore might be selectively influenced by a given gene. Additionally, in our sample, genotypes were not equally distributed in the experimental groups, so that the AA genotype was found in one subject that was in the sham group, while GG genotype was present in four subjects of the real-tACS group and in one subject of the sham group. The AG genotype was present in seven subjects of the real-tACS group and in four subjects of the sham-tACS group. In other words, being the subject with the AA genotype in the sham group, we cannot state whether this genotype might have an effect on the response to tACS. Similarly, the unequal and low number of the other genotypes distributed among the two experimental groups allow us only to speculate that such genotypes do not affect response to tACS in physical performance. Further investigations are needed to understand the nature and interaction between genetic background and NIBS protocol at a cellular and, mostly important, at a behavioral level. A further limitation of the present study is that we cannot draw conclusion on the relationship between the lack of behavioral effects and the excitability levels, since we did not assess motor cortex excitability. Additionally, larger samples or changes in the stimulation setting and in the applied frequency bands are needed to further investigate the effects of tACS in physical performance. Further studies might investigate online effect of tACS applied during physical performance rather than before it. In other words, an ambitious challenge is to assess the efficacy of tACS in real-world sports performance in order to solve the doubt whether tACS supplementation fits into the regulatory framework at the competitive level. In the meantime, it seems likely that tACS will continue to be explored by elite athletes looking for that elusive edge.

## Conclusion

In modern sports, there is huge pressure to improve performance rapidly, both in amateur and professional circles. Therefore, understanding whether neuromodulation through tACS may improve physical performance is a key point of modern sports discipline. This work offers a preliminary and novel first step to study the influence of non-invasive approach with tACS on performance in sports people. Our preliminary results suggest that the modulation of real-life sports performance probably requires further investigation to identify the optimal parameters and stimulation setting. It seems that these settings differ enough from parameters traditionally applied in laboratory context. Further studies are needed to clarify whether tACS might improve real athletes’ performances.

## Data Availability Statement

The raw data supporting the conclusions of this article will be made available by the authors, without undue reservation.

## Ethics Statement

The studies involving human participants were reviewed and approved by the ethic committee of Palermo 1 (4/2020). The patients/participants provided their written informed consent to participate in this study.

## Author Contributions

AG, PP and GB made substantial contributions to conception and design. HM, AI and GM contributed to acquisition of data. SG performed statistical analysis and interpretation of data. AG, GB and PP drafted the article with the help of GM, AI and HM. MO and AP revised the article critically for important intellectual content. All authors approved the final version to be published and agree to be accountable for all aspects of the work in ensuring that questions related to the accuracy and integrity of any part of the work are appropriately investigated and resolved. All authors contributed to the article and approved the submitted version.

## Conflict of Interest

The authors declare that the research was conducted in the absence of any commercial or financial relationships that could be construed as a potential conflict of interest.
